# Bone mineral density measurements derived from dual-layer spectral CT enable opportunistic screening for osteoporosis

**DOI:** 10.1007/s00330-019-06263-z

**Published:** 2019-05-21

**Authors:** Ferdinand Roski, Johannes Hammel, Kai Mei, Thomas Baum, Jan S. Kirschke, Alexis Laugerette, Felix K. Kopp, Jannis Bodden, Daniela Pfeiffer, Franz Pfeiffer, Ernst J. Rummeny, Peter B. Noël, Alexandra S. Gersing, Benedikt J. Schwaiger

**Affiliations:** 1 Department of Radiology, Klinikum rechts der Isar, School of Medicine, Technical University of Munich, Ismaninger Strasse 22, 81675 Munich, Germany; 2 Biomedical Physics & Munich School of BioEngineering, Technical University of Munich, 85748 Garching, Germany; 3 Department of Neuroradiology, Klinikum rechts der Isar, School of Medicine, Technical University of Munich, Ismaninger Strasse 22, 81675 Munich, Germany; 4 Department of Radiology, Perelman School of Medicine, University of Pennsylvania, 3400 Spruce St., 1 Silverstein, Philadelphia, PA 19104 USA

**Keywords:** Bone density, Osteoporosis, Tomography, x-ray computed

## Abstract

**Objective:**

To investigate the in vivo applicability of non-contrast-enhanced hydroxyapatite (HA)-specific bone mineral density (BMD) measurements based on dual-layer CT (DLCT).

**Methods:**

A spine phantom containing three artificial vertebral bodies with known HA densities was measured to obtain spectral data using DLCT and quantitative CT (QCT), simulating different patient positions and grades of obesity. BMD was calculated from virtual monoenergetic images at 50 and 200 keV. HA-specific BMD values of 174 vertebrae in 33 patients (66 ± 18 years; 33% women) were determined in non-contrast routine DLCT and compared with corresponding QCT-based BMD values.

**Results:**

Examining the phantom, HA-specific BMD measurements were on a par with QCT measurements. In vivo measurements revealed strong correlations between DLCT and QCT (*r* = 0.987 [95% confidence interval, 0.963–1.000]; *p* < 0.001) and substantial agreement in a Bland–Altman plot.

**Conclusion:**

DLCT-based HA-specific BMD measurements were comparable with QCT measurements in in vivo analyses. This suggests that opportunistic DLCT-based BMD measurements are an alternative to QCT, without requiring phantoms and specific protocols.

**Key Points:**

*• DLCT-based hydroxyapatite-specific BMD measurements show a substantial agreement with QCT-based BMD measurements in vivo.*

*• DLCT-based hydroxyapatite-specific measurements are on a par with QCT in spine phantom measurements.*

*• Opportunistic DLCT-based BMD measurements may be a feasible alternative for QCT, without requiring dedicated examination protocols or a phantom.*

## Introduction

Fragility fractures are the main symptom of osteoporosis and frequently occur in the thoracic and lumbar spine. Osteoporotic fractures affect the individual patient and are considered to be a relevant public health issue: they substantially contribute to the health care costs [1] and are associated with reduced health-related quality of life [2]. Ultimately, prevalent vertebral and hip fractures lead to an increased risk of mortality for up to 5 and 10 years after fracture event, respectively [3, 4].

Dual-energy x-ray absorptiometry (DXA) and quantitative CT (QCT), the current clinical standards, are known to have limitations such as high susceptibility to confounders like body size or vascular calcifications and limited availability or relatively high radiation doses, respectively [5].

Furthermore, these methods are under-used with participation rates for DXA of only 30% and 4% in eligible women and men over 65 years, respectively [6]. Consequently, opportunistic imaging is particularly promising for spinal osteodensitometry, as many patients undergo diagnostic CT of the chest or abdomen for a variety of indications and these scans mostly also include the spinal column [7, 8]. Since osteoporosis is considered to be an underdiagnosed and undertreated condition [1], opportunistic screening would enhance the identification of individuals with low BMD being at risk for spinal fractures and thus would enable the prevention of major fragility fractures by an early initiation of therapy, e.g., with pharmacological treatment.

For material-specific measurements and other applications, dual-layer CT (DLCT) has gained growing attention: This special approach of dual-energy CT (DECT) was only recently introduced in clinical routine and uses two scintillator elements, one superimposed on the other, coupled to a photodiode. Both layers have sensitivity maxima in two different parts of the x-ray spectrum and therefore provide spectral information [9]. This information can be used to decompose attenuation values and derive material-specific density information, thus combining volumetric measurements specifically for calcium hydroxyapatite (HA) with a morphological assessment [10].

Of note, the basic concept of dual-energy imaging is not a new one in osteodensitometry. Dual-energy techniques have been introduced more than 30 years ago, both in the context of projectional radiography [11–13] and computed tomography [14, 15]. Especially for DECT, an early application in bone mineral quantification was to reduce measuring inaccuracies of conventional single-energy CT that are caused by beam hardening artifacts or vertebral bone marrow fat [16, 17].

Other current approaches to DECT imaging, acquiring spectral information, are dual-source CT (DSCT) and single-source CT with fast kV-switching. While the former uses two x-ray sources and two detectors in a nearly perpendicular setup, the latter employs one x-ray source with rapid tube voltage switching and one detector [10].

Contrary to these source-based DECT setups, DLCT only requires one x-ray source and both detector layers are always “ON” in all examinations. Therefore, dual-energy information is available from every routine clinical examination whereas in contrast, in dual-source or rapid kV-switching systems, examinations are often performed in single-energy mode only, and dual-energy information are collected only if prescribed prior to the examination. Due to its simultaneous data acquisition of the low- and high-energy data sets, DLCT, however, continuously allows for additional analyses in already-acquired imaging data such as BMD measurements in non-dedicated examinations.

This opportunistic approach could help reduce the gap in the detection of patients with low BMD and consequently pave the way for establishing appropriate treatment. The first ex vivo studies, in which HA-specific BMD measurements were assessed in hydroxyapatite-containing phantoms and vertebral specimens, demonstrated the high accuracy of HA-specific BMD measurements [18, 19].

Therefore, the purpose of this study was to investigate the clinical applicability of non-contrast-enhanced DLCT-based HA-specific BMD quantification in vivo by evaluating its accuracy compared with phantom-calibrated QCT-based BMD measurements.

## Methods

### Ex vivo calibration and measurements in a standardized phantom

CT images were acquired with one DLCT scanner (IQon Spectral CT, Philips Healthcare).

A standardized spine phantom (European Spine Phantom (ESP), serial number ESP-040, QRM GmbH) consisting of water-equivalent plastic and HA inserts simulating trabecular bone densities of 50.0 (HA50), 98.4 (HA100), and 197.6 (HA200) mg/ml HA was measured. It should be noted that the exact HA densities as specified by the manufacturer were used for all calculations and analyses, whereas the nominal values were used only for illustrational purposes in tables and figures. The ESP is a tool for standard quality control in DXA and QCT [20–22]. To determine the precise spectral absorption behavior of the ESP, an ultra-high-dose examination protocol with a fixed tube voltage of 120 kVp and an exposure of 1000 mAs was used. Spectral base image (SBI) data was reconstructed using a standard bone filter kernel (YB) with axial slice thickness of 0.9 mm. The measured data was averaged over cylindrical regions of interest (ROIs) of 10.0 mm (height) × 100 mm^2^ (base area) to reduce noise.

SBI data contains information on energy-dependent attenuation behavior, extracted with the use of dual-layer detector technology [23]. This information can be used to create virtual monochromatic (MonoE) images, which are equivalent to images acquired with a monoenergetic x-ray source [24]. These images were generated with IntelliSpace Portal 10.1.0 (Philips Healthcare).

In addition to the calibration scan, 18 scans were performed using different scan parameter combinations with the following variations in patient size, table height, and exposure, to simulate various patient setups:

- Fat-equivalent extension rings (QRM GmbH) (for the simulation of different degrees of obesity): no ring, ring size S, and ring size M (50 mm and 100 mm thick fat-equivalent ring, respectively)
- Centered and off-centered (patient positioning table was moved 43 mm away from the field of view center) table positions
- 50, 100, and 200 mAs exposure
- Tube voltage of 120 kVp

CT numbers from monoenergetic images at 50 and 200 keV were used for BMD quantification: Projection points along the optimal regression line are calculated by applying a projection with an angle of 32° to the calibration line to all scan setups. Via the known BMD values of the ESP, every point on the calibration line can be assigned to a specific BMD using the linear relation

$$ BMD\ \left[\frac{mg}{ml}\right]= Mono{E}_{projection}(50)\ \left[ HU\right]\times u+v. $$

*u* and *v* are the slope and intercept of a linear regression between the MonoE(50) values of the calibration scan and the manufacturer-specified BMDs. Finally, for comparison purposes, the ESP scans as specified above were repeated using routine QCT examination parameters. QCT-based BMD values were calculated using a clinical QCT phantom and its corresponding software (QCT Pro, Mindways Software, Inc.).

### Patient cohort for in vivo BMD measurements

Institutional Review Board approval was obtained previously to this study (Ethics Commission of the Medical Faculty, Technical University of Munich, Germany). Written informed consent was waived for this retrospective analysis of routinely acquired imaging data.

In our institutional PACS, patients who underwent a QCT examination on the DLCT scanner between November 2016 and February 2018 were retrospectively identified. All examination data had to contain spectral information, and the thoracolumbar spine had to be examined in the presence of a standardized QCT-phantom as specified above. Patients with intravenous contrast (*n* = 39) or metal implants in the thoracolumbar spine or close vicinity (e.g., spondylodeses, aortic stent grafts; *n* = 36) were excluded. A total number of *n* = 33 subjects (mean ± SD; 66 ± 18 years; 11 women, 57 ± 22 years; 22 men, 69 ± 13 years) were used for the comparison of DLCT- and QCT-based BMD measurements with a total of 174 vertebral bodies.

### DLCT imaging protocols and post-processing

All CT images were acquired with the same DLCT scanner as used for phantom measurements during clinical routine.

Routine protocols with tube voltage fixed at 120 kVp were used for all scans. Here, patient exposures varied between 20 and 294 mAs, with a mean exposure of 84 ± 61 mAs (mean ± SD). Mean CT dose indices (CTDI_vol_) were 7.7 ± 5.5 mGy over all patients. SBI data sets were reconstructed using standard and bone filter kernels (B, YB, YC), with a slice thickness of 0.9 mm. The distance between the top of the patient table and the center of rotation was 151 mm ± 34 mm.

**Fig. 1:**
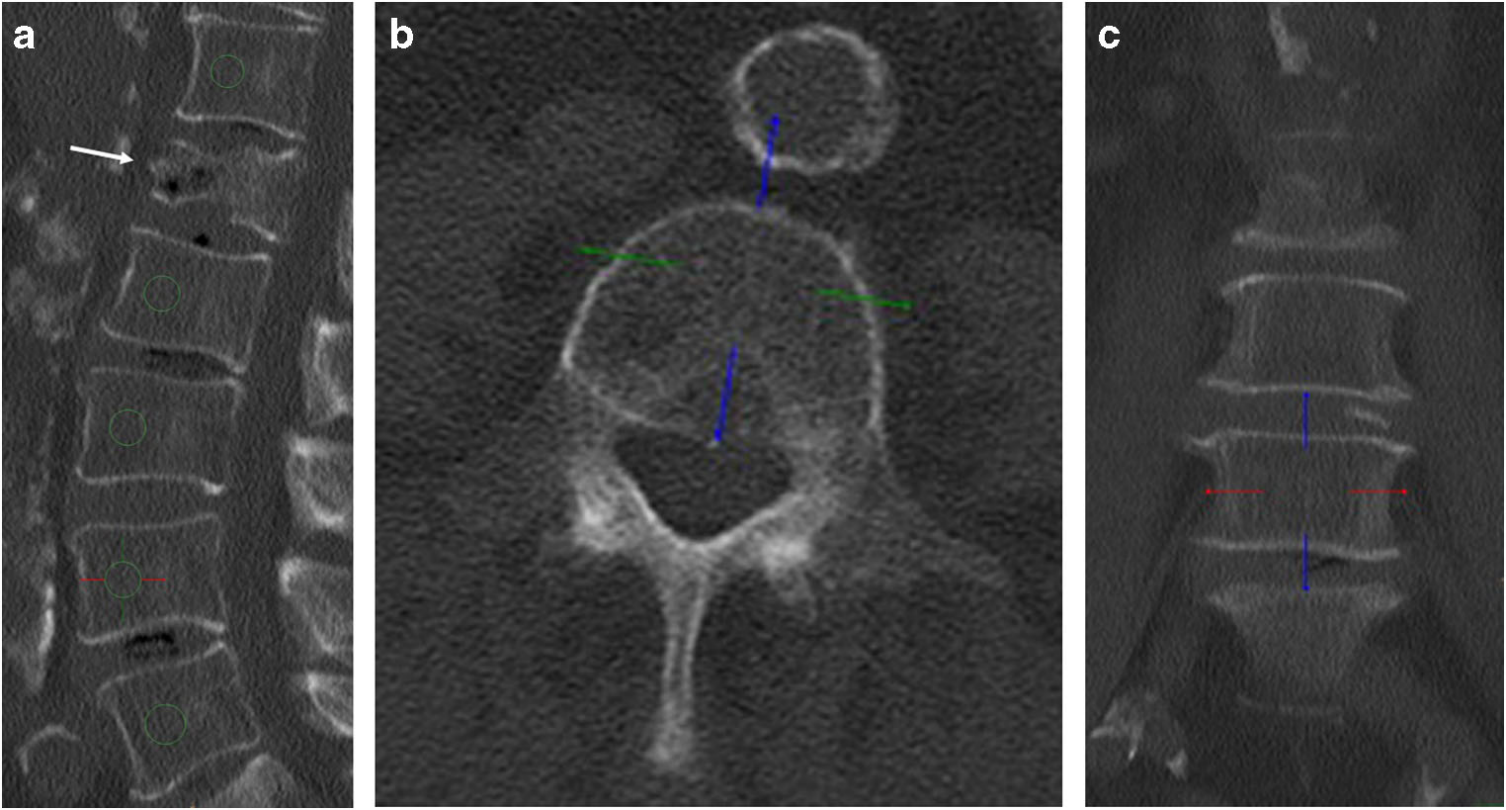
Sagittal (**a**), axial (**b**), and coronal (**c**) reformation of dual-layer spectral CT imaging of a 77-year-old male patient after history of falling. ROIs are positioned in ventral halves of T12 and L2–L5; L1 was excluded due to a compression fracture (white arrow)

Under the supervision of a radiologist with 7 years of experience in spine imaging (BJS), a trained researcher (FR) manually placed circular ROIs onto sagittal reformations (slice thickness, 10 mm) of the thoracolumbar spine as these reformations allow best for synchronous morphological assessment including fractures that may be missed in original axial imaging data (e.g., height loss of vertebrae). ROIs had a diameter of exactly one-third of the particular vertebral body’s height and were positioned in the ventral halves of the trabecular compartment of the vertebrae (Fig. 1). On average, five vertebrae per patient were measured (preferably T12, L1, and L2; range T10 to L5). Vertebral bodies with substantial degenerative changes (e.g., severe osteochondrosis with adjacent sclerosis), fractures, or other pathologies such as hemangioma were excluded and the BMD measurements were consequently obtained from the adjacent vertebrae that were not excluded due to a pathology.

Mean CT numbers for both conventional and corresponding virtual monochromatic images at 50 and 200 keV were extracted from ROIs. HA-specific BMD quantification of all patients was computed using reconstructions of virtual monochromatic images at 50 and 200 keV as described for phantom measurements. For comparison, BMD assessment was also performed by using the QCT Pro calibration phantom (Mindways Software, Inc.) with BMD being calculated in accordance with the conventional QCT method and clinical standards.

### Statistical analysis

For the inter-method comparison of ex vivo phantom measurements, means of differences between the scan and manufacturer-specified BMDs were assessed for DLCT versus QCT scans with identical examination parameters, respectively, using a two-sided paired samples *t* test.

On a vertebral-body-base, Pearson’s *r* was determined to assess the correlation of DLCT- and QCT-based BMD. A Bland–Altman plot was used to evaluate the agreement of both measurements [25].

To assess the intrareader agreement of HA-specific BMD measurements, the same researcher repeated measurements in 20% of the vertebral bodies (*n* = 35) after 4 months, blinded for previous results, and two-way mixed intraclass correlation coefficients (ICC) were calculated.

Statistical analyses were performed with SPSS 25 (IBM). A two-sided *p* value of less than 0.05 was considered to indicate a statistically significant difference.

## Results

### Ex vivo DLCT-based phantom measurements

**Fig. 2:**
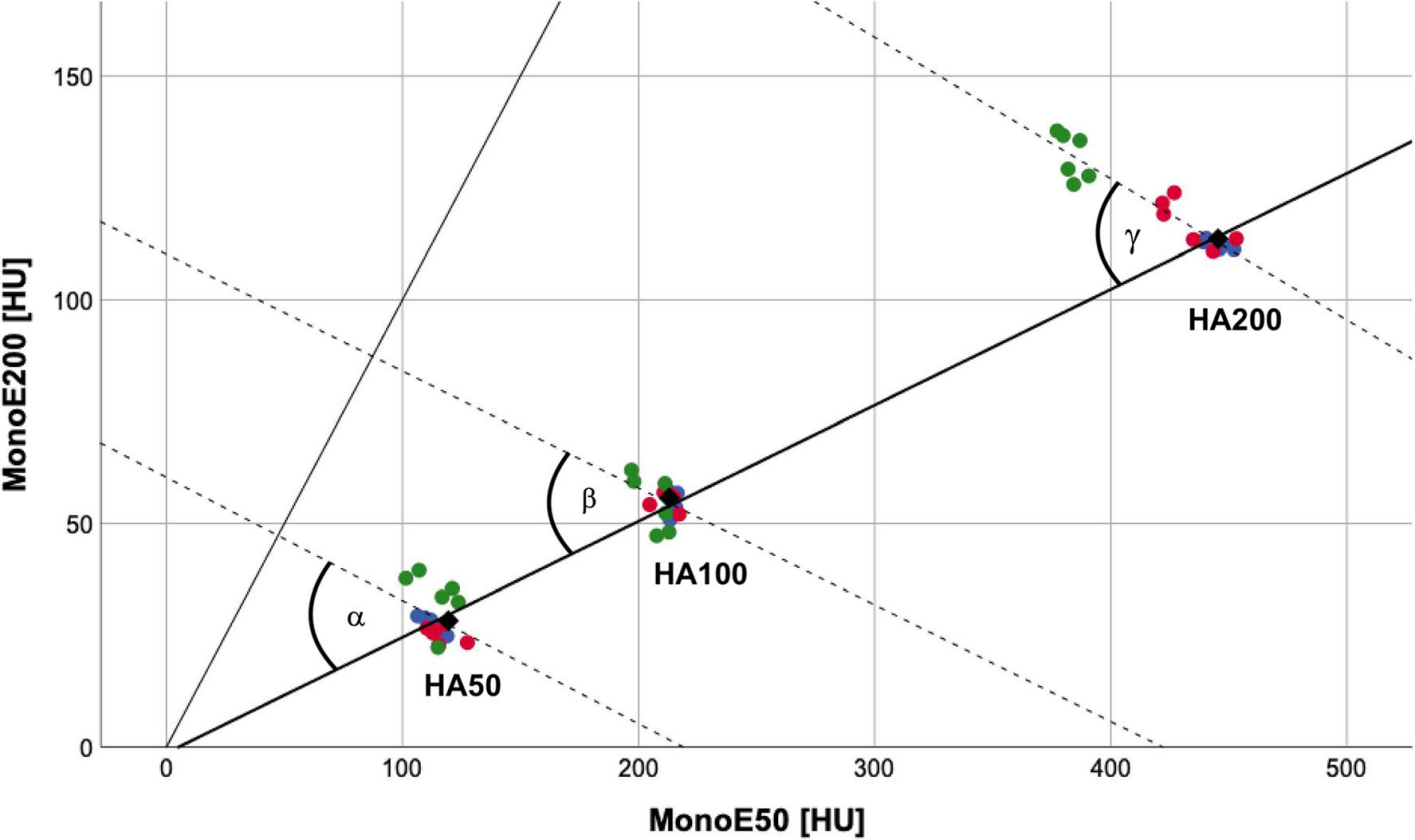
Scatter plot showing phantom measurement results of calibration scans with different simulated setups in terms of patient size (blue represents “no extension ring”, red represents ring size S, green represents ring size M), table position, and exposure for three artificial vertebral bodies of prespecified HA densities (50.0 (HA50), 98.4 (HA100), and 197.6 (HA200) mg/ml HA). The thick black line represents the regression line of the ultra-high-dose calibration scan (1000 mAs exposure), the thin black line represents the bisecting line, and broken lines are the regression lines for measurements of individual HA inserts. Projection angles are *α* = 29.9°, *β* = 29.1°, and *γ* = 32.0°

The spectral data obtained from the calibration scan and the scans simulating different patient setups were plotted separately for three artificial vertebral bodies (Fig. 2), and a projection calibration was executed, defining projection angles of *α* = 29.9°, *β* = 29.1°, and *γ* = 32.0° for vertebral bodies containing 50.0, 98.4, and 197.6 mg/ml HA, respectively. These projection angles represent the angles between the linear regression line of the high-dose calibration scan for different BMDs and the linear regression of the 18 scans in different setups for each BMD. Deviations from the calibration line are mainly caused by different patient sizes, while different table heights and exposure only cause minor aberrations. For our algorithm, an angle of *γ* = 32.0° was used, as this projection calibration sets the highest correlation coefficient (*r* = − 0.947).

In phantom measurements, means of differences between BMD scan results and manufacturer-specified values averaged for all different scan settings tended to be lower for DLCT (3.9 mg/ml HA) compared with those of QCT (4.8 mg/ml HA) (Table 1), although this difference was not statistically significant (*p* = 0.152).

**Table 1:** Differences between measurements (QCT/DLCT) and manufacturer-specified BMD. Means of differences (MOD) are calculated from the absolute deviations from manufacturer’s specifications of 50.0, 98.4, and 197.6 mg/ml HA densities (HA50, HA100, HA200, respectively)

Ring size	Table position	Exposure (mAs)	QCT			DLCT			MOD (QCT)	MOD (DLCT)
			HA50	HA100	HA200	HA50	HA100	HA200		
No ring	Centered	50	47.3	95.6	201.6	46.8	93.0	194.7	3.2	3.8
		100	47.3	98.6	201.4	46.6	94.6	196.0	2.2	2.9
		200	47.3	96.6	199.6	47.7	95.1	195.9	2.2	2.4
	Off-centered	50	45.3	96.2	203.3	47.9	90.2	194.1	4.2	4.6
		100	50.3	96.4	199.0	47.4	95.6	194.4	1.2	2.9
		200	46.1	94.4	200.5	47.2	94.0	195.0	3.6	3.3
S	Centered	50	52.9	99.5	199.7	46.1	90.8	193.5	2.0	5.2
		100	45.7	97.5	196.2	45.9	94.6	198.1	2.2	2.8
		200	46.0	94.5	199.8	48.7	94.3	196.6	3.4	2.1
	Off-centered	50	49.0	94.5	195.4	44.3	92.2	193.5	2.4	5.3
		100	49.1	96.2	201.1	47.8	94.5	199.7	2.2	2.7
		200	46.3	94.8	194.7	46.3	94.4	194.8	3.4	3.5
M	Centered	50	54.5	93.7	192.3	53.9	86.1	198.1	4.8	5.6
		100	45.6	104.4	190.3	55.6	93.2	199.0	5.9	4.1
		200	46.3	97.1	189.9	53.1	94.9	198.3	4.2	2.4
	Off-centered	50	40.9	88.8	179.6	43.9	88.0	190.7	12.2	7.8
		100	35.4	86.8	179.0	52.6	91.0	193.7	14.9	4.6
		200	41.3	87.1	182.5	55.3	96.1	192.7	11.7	4.2
Overall			46.5	95.2	194.8	48.7	92.9	195.5	4.8	3.9

Explicit results and means of differences between measurements and manufacturer’s specifications (MOD) given in mg/ml

### In vivo DLCT-based BMD measurements

**Fig. 3:**
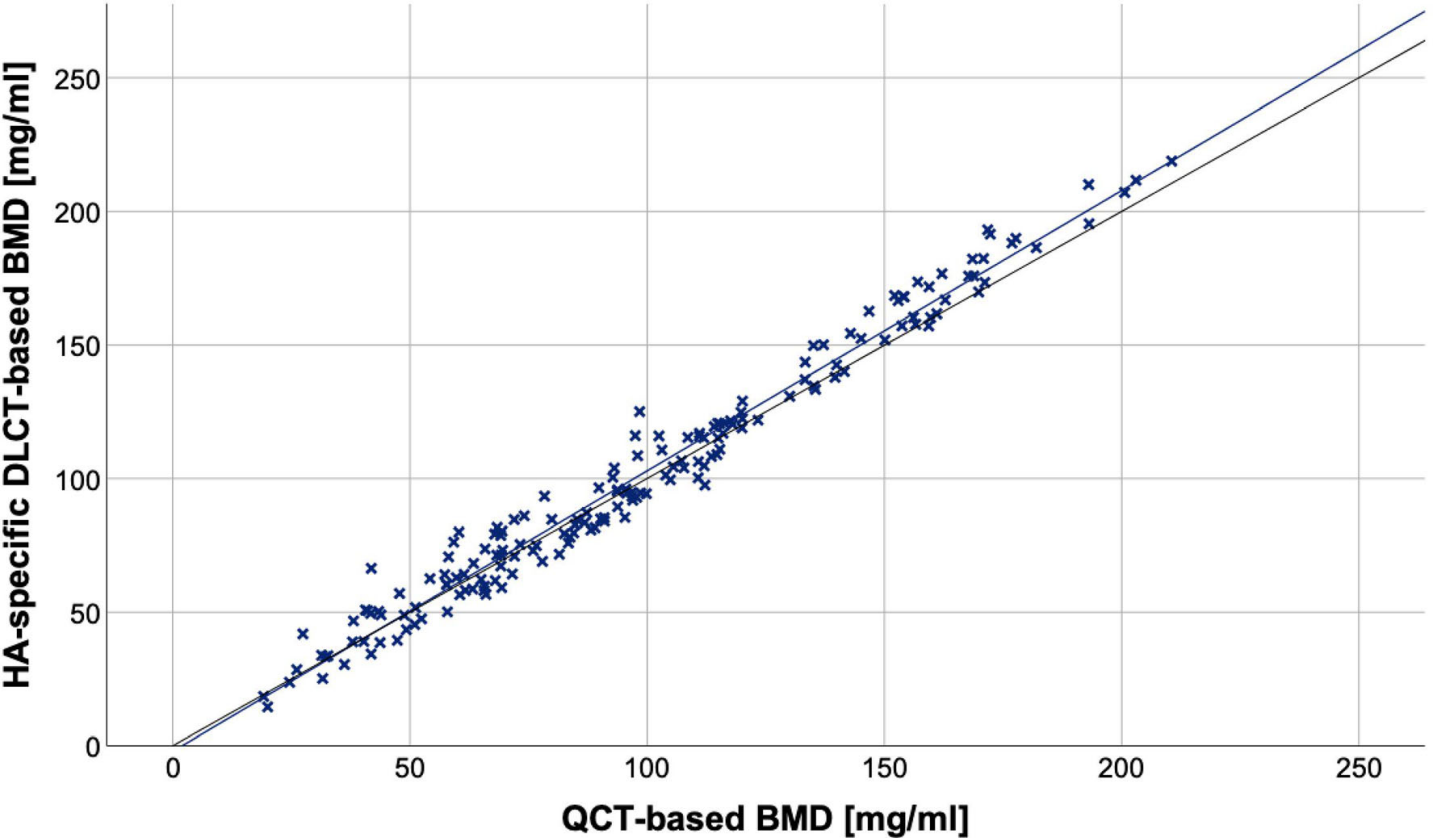
Scatter plot showing BMD values of 174 vertebral bodies from 33 patients, BMD values obtained by phantomless DLCT (*y*-axis) and phantom-based QCT (*x*-axis); the blue line represents the linear regression line, and the black line represents the bisecting line. A significant correlation between both measurement methods can be identified (*r* = 0.987 [95% confidence interval, 0.963–1.000]; *p* < 0.001)

**Fig. 4:**
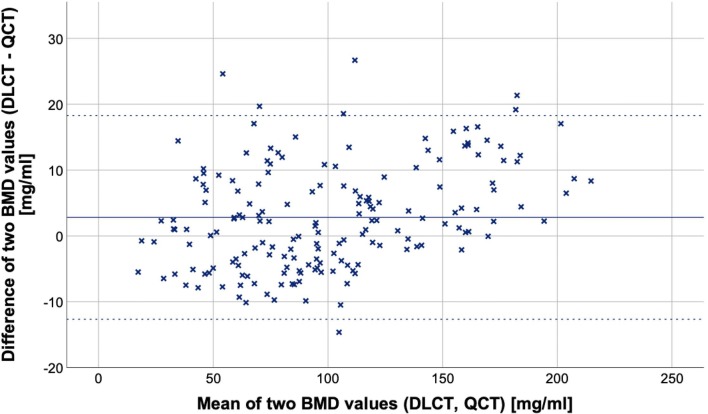
Bland–Altman plot showing data of 174 vertebral bodies from 33 patients, solid line indicating the mean BMD difference, dotted lines indicating the 95% limits of agreement (mean difference ± 1.96 SD). The plot shows means of DLCT- and QCT-based BMD values on the *x*-axis and differences of both measurements (DLCT- minus QCT-based BMD) on the *y*-axis. The mean difference is $\overline{d}$= 2.81 mg/ml (95% confidence interval, 1.64–3.99 mg/ml) with a standard deviation of differences of *s*_d_ = 7.89 mg/ml. The 95% limits of agreement subsequently are − 12.66 mg/ml and + 18.29 mg/ml. This indicates a substantial agreement between both measurement methods

BMD values derived from the DLCT-based method and from QCT were highly correlated (*r* = 0.987 [95% confidence interval, 0.963–1.000]; *p* < 0.001; Fig. 3). The corresponding Bland–Altman plot shows a substantial agreement between both methods (Fig. 4). DLCT-based BMD measurements show a mean difference from QCT measurements of $\overline{d}$= 2.81 mg/ml (95% confidence interval, 1.64–3.99 mg/ml) with a standard deviation of differences of *s*_d_ = 7.89 mg/ml. The 95% limits of agreement subsequently are − 12.66 mg/ml and + 18.29 mg/ml.

The intrareader agreement for DLCT-based BMD measurements was excellent (ICC, 0.997 [95% CI, 0.994–0.998]).

## Discussion

In this study, opportunistic HA-specific BMD measurements derived from clinical DLCT examinations showed an excellent correlation with QCT-based BMD measurements. Furthermore, measurements in the standardized ESP suggest that the presented DLCT-based method is on a par with those from QCT.

In a clinical assessment, the American College of Radiology lists the following BMD ranges for use to approximately assign QCT-based BMD to the WHO diagnostic categories “osteopenia” and “osteoporosis”, which are actually exclusively established for DXA measurements: for osteopenia, BMD values range between 80 and 120 mg/ml and for osteoporosis, BMD values are < 80 mg/ml [26]. In this clinically relevant range, both methods showed a substantial agreement in in vivo measurements. Here, a slight but significant systematic difference can be observed with DLCT consistently yielding higher BMD than QCT. Larger deviations between the two methods at higher BMD values mainly result from the introduced projection angle, taking the influence of simulated abdominal fat tissue into consideration. Of note, over all simulated measurement setups, phantom measurements deviated less from known HA densities in the ESP for the DLCT-based method than for QCT.

This may be caused by the so-called fat error affecting non-HA-specific QCT measurements: several studies have identified a high bone marrow fat fraction as a source of error in osteodensitometry, which is associated with an underestimation of BMD in QCT-based measurements by using magnetic resonance spectroscopy [27, 28] or chemical analysis [29, 30] as standard of reference. Of note, the determination of ash density values in vertebral specimens demonstrated a reduction of the fat-related error with DECT [30].

In a different DECT approach, Booz et al showed the feasibility of phantomless in vivo BMD assessment using DSCT [31]. DSCT, however, has inherent limitations compared with the dual-layer setup used in this study. The nearly perpendicular arrangement of both x-ray sources is not only responsible for an asynchronous detection of congruent projections and therefore does not allow material decomposition in projection space, but it is also the cause of scatter radiation on respective radiation fields with the need for correction [10].

The method for DLCT-based BMD quantification as used in this study is similar to a previous ex vivo study by Mei et al [19]. In this previous study, different degrees of obesity were not found to influence HA-specific BMD measurements significantly within the range of clinical examination parameters. While differences between DLCT-based measurements and known HA densities of the ESP in suboptimal examination setups were still within acceptable dimensions, we decided to additionally introduce a projection calibration step as described above to further increase accuracy of measurements. Of note, this step only needs to be performed once on the CT scanner after measuring the ESP.

DLCT data is acquired without preselected examination protocols and can therefore be used for different opportunistic analyses, e.g., to reduce artifacts from metallic hardware [32] or to create virtual non-contrast images from contrast-enhanced examinations [33]. In the detection of low BMD, opportunistic use of already-available imaging data has recently received a lot of attention [7, 8, 34–36] for several reasons: Many patients at risk for fractures due to impaired bone stability undergo CT imaging for other reasons such as vertebral fracture assessment in patients with primary osteoporosis, but also CT staging examinations e.g. in the presence of malignant conditions. Using these images for BMD analyses would provide an accurate biomarker without additional radiation doses, examination time, and costs, thus potentially closing the diagnostic gap for individuals at increased fracture risk.

This study has limitations. First, the patient population was rather small since for the inter-method comparison, DLCT and QCT examinations performed on the same scanner were required for this study population. In addition, strict exclusion criteria regarding patients with metal implants in adjacent structures (e.g., spondylodeses, aortic stent grafts) were applied to avoid possible measurement errors by reason of beam hardening artifacts. Comparison of both CT techniques in quantifying bone mineral density was therefore performed on a base of single vertebral bodies (*n* = 174). As measurements in the standardized phantom suggest, the results of the DLCT-based method tally with those obtained by QCT. However, before final conclusions are drawn, both methods should be objectively compared using a third method as standard of reference. This could be determining the BMD with both methods in vertebral specimens which are then burned and chemically analyzed for their HA density, which, however, is not applicable in vivo.

Besides, BMD has shown to be only one of several parameters for fracture risk evaluation, with trabecular bone microstructure being another relevant factor. Whether microstructure assessment based on DLCT and QCT may generate different results should also be assessed in the future. Finally, in this analysis, only non-contrast-enhanced CT examinations were included. Since many clinical examinations are performed with intravenous contrast (e.g., staging examinations), the feasibility of measurements in those contrast-enhanced examinations should be investigated in future studies.

In conclusion, opportunistic HA-specific BMD measurements derived from clinical DLCT examinations were highly correlated and showed a substantial agreement with QCT-based BMD measurements. Moreover, phantom measurements suggest that the presented DLCT-based method is on a par with QCT. This suggests that opportunistic HA-specific measurements may be an adequate alternative for early detecting patients with low bone mineral density in clinical routine and may support optimal individual therapeutic decisions.

## Abbreviations

BMD: Bone mineral density
DECT: Dual-energy computed tomography
DLCT: Dual-layer computed tomography
DSCT: Dual-source computed tomography
DXA: Dual-energy x-ray absorptiometry
ESP: European Spine Phantom
HA: Hydroxyapatite
MonoE: Virtual monoenergetic
QCT: Quantitative CT
SBI: Spectral base image
